# Optically Controlled TiO_2_-Embedded Supercapacitors: The Effects of Colloidal Size, Light Wavelength, and Intensity on the Cells’ Performance

**DOI:** 10.3390/nano12111835

**Published:** 2022-05-27

**Authors:** Haim Grebel, Tazima Chowdhury

**Affiliations:** The Center for Energy Efficiency, Resilience and Innovation (CEERI), The Electronic Imaging Center (EIC), The New Jersey Institute of Technology (NJIT), Newark, NJ 07102, USA; tsc9@njit.edu

**Keywords:** supercapacitors, active-carbon films, semiconductor embedded supercapacitors, particles and colloidal embedded films

## Abstract

Optically controlled supercapacitors (S-C) could be of interest to the sensor community, as well as set the stage for novel optoelectronic charging devices. Here, structures constructed of two parallel transparent current collectors (indium-tin-oxide, ITO films on glass substrates) were considered. Active-carbon (A-C) films were used as electrodes. Two sets of electrodes were used: as-is electrodes that were used as the reference and electrodes that were embedded with submicron- or micron-sized titanium oxide (TiO_2_) colloids. While immersed in a 1 M Na_2_SO_4_, the electrodes exhibited minimal thermal effects (<3 °C) throughout the course of experiments). The optically induced capacitance increase for TiO_2_-embedded S-C was large of the order of 30%, whereas S-C without the TiO_2_ colloids exhibited minimal optically related effects (<3%). Spectrally, the blue spectral band had a relatively larger impact on the light-induced effects. A lingering polarization effect that increased the cell capacitance in the dark after prolonged light exposure is noted; that effect occurred without an indication of a chemical reaction.

## 1. Introduction

There is growing interest in supercapacitors (S-C) due to the demand for clean and renewable energy. S-C are short-term energy storage elements that exhibit high-power density (namely, are able to quickly charge and discharge), good reversibility, and a long life cycle [1,2,3]. S-C are used in a wide range of applications, such as consumer electronic products, memory backup devices, hybrid electric vehicles, and power supply systems [4,5]. They were proposed as buffers to highly fluctuating power grids that are interfaced with sustainable sources [6]. Low-energy density (the amount of energy that is actually stored in them) is a major drawback that limits the use of S-C.

Titanium dioxide (TiO_2_) is a large bandgap semiconductor material that is also used as a photocatalyst [7]. Integrating it with S-C [8,9,10,11,12,13,14,15,16,17,18] or dye-sensitized solar cells [19] has been reported. In order to improve its performance, TiO_2_ nanocrystallines have been synthesized with carbon-based materials or with highly conducting conjugated polymers [10,11,12,15]. Due to its catalytic effect, a typical TiO_2_-based S-C exhibits a combined pseudocapacitance and electric double-layer characteristics.

Here, we study the light-induced capacitance increase when incorporating TiO_2_ colloids in the active-carbon-(A-C)-based electrodes of S-C. This capacitance increase was studied as a function of light intensity, irradiation wavelength, and colloidal size. In comparing 2- and 3-electrode set-ups, more attention was given to the 3-electrode set-up because thermal effects are largely suppressed when immersing the electrode in an electrolyte.

## 2. Materials and Methods

**Figure 1:**
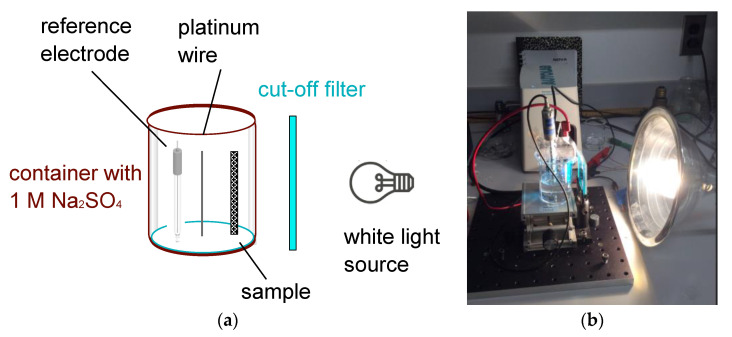
(**a**) Schematics of the 3-electrode setup. (**b**) Image of 3-electrode experimental setup. A white-light source illuminates the sample through a color (here, blue) filter. The electrode was immersed in an electrolyte (1 M Na_2_SO_4_) and a comparison was made between the illuminated and non-illuminated cases. The Metrohm measurement system (white box) is shown in the back, in front of the lab notebook.

The S-C electrodes were made of active-carbon powder (A-C). The binder was poly(methyl methacrylate), PMMA. The slurry was drop-casted on glass substrates that were coated with transparent and conductive films of indium tin oxide (ITO; average sheet resistance Rsqr = 6 Ω/□ and average thickness of 220 nm, made by Huanyu, Yueqing, China). Studies were made on submicron-sized titanium dioxide (TiO_2_, size 0.2 µm, rutile, Polyscience), and on micron-sized titanium (IV) dioxide (2 µm, metal basis, Alfa). In making the slurry, 6 mg of the binder and PMMA powder (Polyscience, Niles, IL, USA) were first dissolved in 1 mL of toluene at 70 °C for 20 min on a hot plate. Then, 10 mg of TiO_2_ powder of either particle size was added to the solution and the batch was sonicated for 1 h using a horn antenna. An amount of 100 mg of powdered A-C (specific surface area of 1100 m^2^/g, produced by General Carbon Company, GCC, Paterson, NJ, USA) was added and mixed with the other components for an additional 2 h. The slurry was then drop-casted on precut and cleaned ITO-coated glass substrates (the current collectors) and let dry on a hotplate at 90 °C for a few minutes. The thickness of the films varied; most TiO_2_-embedded films were rather thick, of the order of 450 µm, but a few films were thin, of the order of 150 µm. The films’ thicknesses were much larger than the light penetration depths as was visibly observed. Since an effective value of the cell capacitance depends, among other things, on the film’s area, thickness, its contacts and the current collectors’ resistance, we used a relative capacitance measure (C_light ON_ − C_light OFF_)/C_light OFF_) rather than the capacitance values themselves. This measure underestimates the effect of light since the light absorption occurs in a narrow region, narrower than the film thickness. Although initially it was thought to lay out a thick film of A-C to avoid the light impact on the ITO at the film’s back, it was found that the results did not depend much on whether the ITO was facing the optical source or away from it. Therefore, and in order to make better comparisons between the 3-electrode and the 2-electrode setups, all data presented here were taken with the ITO electrode facing the light source. An amount of 1 M of sodium sulfate (Na_2_SO_4_) was used as an electrolyte and platinum wire was used as the counter electrode. The experimental 3-electrode setup is shown in Figure 1a and pictured in Figure 1b.

Electrochemical measurements were performed with a Potentiostat/Galvanostat (Metrohm, Herisau, Switzerland) in mostly a 3-electrode configuration. The samples were illuminated at two different intensities using 75 W and 300 W incandescent light bulbs, which translates to light intensities of 45 mW/cm^2^ and 180 mW/cm^2^, respectively, at a distance of 20 cm from the sample. These numbers are for dry samples only and do not include the light absorption by water (which varies substantially between the blue at 450 nm and the near IR at 800 nm). At the same time, one should note that the maximal light absorption by water at the near infra-red (at 800 nm) is of the order of 0.01 1/cm which translates to a very small absorption in our setting; the distance between the container’s wall and the sample was <1 cm, hence the total absorption was <0.01.

The white-light intensity of the source as measured at the sample surface was assessed by using a bolometer, situated at the same distance as the sample. In order to sort the absorption of the sample at various incident wavelengths, we used two optical filters: a short wavelength (blue) filter that transmits ca 10% of the total white-light intensity in the range of 420 to 550 nm; a long wavelength (yellow) filter that transmits 40% of the white-light intensity in the range above 550 nm. Note that the incandescent white-light source emits wavelengths up to 10 µm and beyond.

## 3. Results

**Figure 2:**
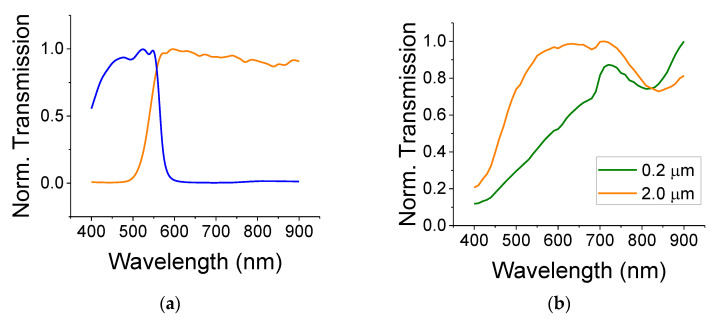
(**a**) Normalized transmission through the optical filters used in the experiments: a short-pass wavelength (blue) and long-pass wavelength (yellow) filters. (**b**) Normalized transmission through films composed of the smaller (0.2 µm) and larger (2 µm) particles of TiO_2_. The particles were embedded in a thin PMMA matrix.

The normalized optical transmission curves for the colored filters are shown in Figure 2a. The filters are denoted as short-pass (blue curve)—transmitting in the range of 420–550 nm—and long-pass (yellow curve)—transmitting above 550 nm. The normalized optical transmission for the TiO_2_ colloids (small, 0.2 µm and large, 2 µm that are embedded in poly(methyl methacrylate), PMMA) are shown in Figure 2b for dry films (see Ref. [20] regarding light absorption by wet films). Each transmission curve was first referenced to its substrate (a glass slide) in order to remove the effects of the glass (if any). Then, the curve was normalized to its peak transmission because our interest is in finding its spectral dependence rather than the film loss (which also depends on the film thickness and scattering). The glass slide showed a fairly constant transmission throughout the spectral range between 400 nm and 900 nm. The thin ITO film transmission data are flat throughout the visible spectra with a small absorption near the blue region of 400 nm. The binder, PMMA, also exhibited a flat spectral response in the visible range with a transmission coefficient of 0.9. As can be seen from Figure 2b, the larger TiO_2_, exhibited a strong absorption (transmission dip) in the blue/green region. This is consistent with a bandgap of ca 3 eV. The small TiO_2_ particles exhibited an additional large scattering in the spectral region between 400 and 700 nm. The bandgap of 0.2 µm particles should differ only a little from the bandgap of the larger particles of 2 µm—quantum-sized effects usually appear at much smaller sizes of ca 2–10 nm. Figure 2b also exhibits an absorption line (transmission dip) at 840 nm that could be attributed to water.

**Figure 3:**
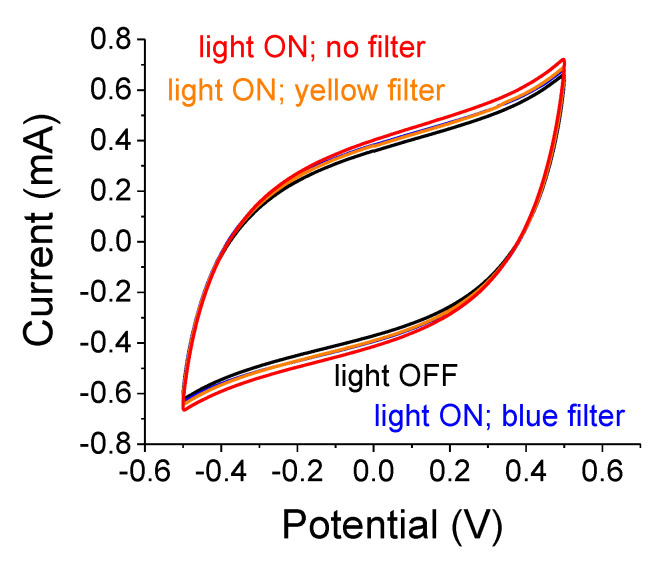
Cycle-voltammetry (C-V) for 0.2 µm particles embedded in A-C/PMMA using a 2-electrode setup. The S-C was illuminated by a 75 W white-light source. The cases with no filter (red), blue, and yellow filters are shown. The scan rate was 0.1 V/s.

Preliminary experiments with a 2-electrode setup and a 75 W white-light source are shown in Figure 3. The largest effect is obtained without any color filter due to a larger irradiation intensity and a wider blue band spectrum. The light-induced capacitance increase is similar when using the blue or the yellow filters despite the large differences in their respective illumination intensities; the intensity of the blue band is one-quarter of the intensity that is transmitted through the yellow filter. Despite the catalytic characteristics of TiO_2_, there were no noticeable reaction peaks. As we will comment later, the samples exhibited an increasing polarization effect following a prolonged light exposure. That effect persisted in the dark for over 5 min after switching off the illuminator. Unlike SiC-embedded [20] or n-Si-embedded A-C [21], one needs to remeasure the sample in the dark after each light exposure in order to maintain a better baseline.

A summary of the relative capacitance changes, (C_light ON_−C_light OFF_)/C_light OFF,_ when the samples were illuminated by a 75 W white-light source through the various filters is provided in Table 1. The capacitance increase for the filtered light is small and depends on the light intensity. Also, note that the blue and yellow filters resulted in similar effects as those alluded to in Figure 3.

**Figure 4:**
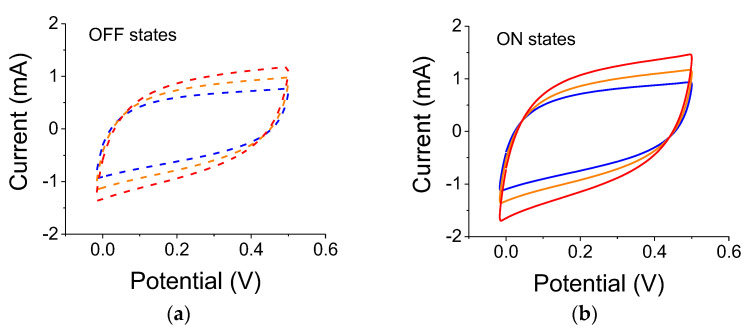
(**a**) Light OFF, C-V reference curves for 0.2 µm particles measured before each illumination by a 300 W white-light source. The colored dashed curves correspond to successive references for data taken with the blue, yellow, and no filter, respectively. (**b**) C-V curves for light ON cases; the blue, yellow, and red curves correspond to illumination with blue, yellow, and no filter, respectively.

Summaries of all C-V results are provided in Table 2 and Table 3 below. One ought to look at the trends (blue vs. yellow filters, intensity-related, scan rates, etc.). The results from the 3-electrode set-up are shown in Figure 4 for samples illuminated by a 300 W lamp. As clearly seen, the reference capacitance under dark conditions increased after each light exposure. The experiments were conducted using filters in the order of blue, yellow, and no filter. Although there is a lingering polarization effect exhibited by the successive measurements in light-OFF conditions (OFF states), there is also a clear light-induced capacitance increase (ON states). The relative capacitance values for a scan rate of 0.01 V/s were 21%, 18.5%, and 23.5% for blue, yellow, and no filter, respectively.

**Figure 5:**
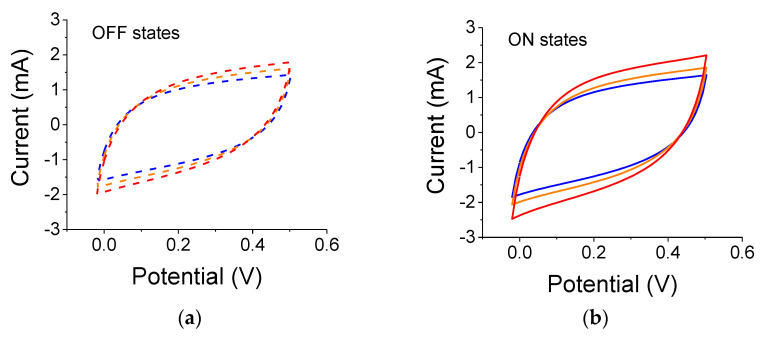
(**a**) Light OFF, C-V reference curves for 0.2 µm particles measured before each illumination by a 75 W white-light source. Experiments were carried out after the conclusions of the experiments with the 300 W lamp. The colored dashed curves correspond to successive references for data taken with the blue, yellow, and no filters, respectively. (**b**) C-V curves for light ON cases; the blue, yellow, and red curves correspond to illumination with blue, yellow, and no filter, respectively. Scan rate was 0.01 V/s.

The results for samples illuminated by a 75 W white-light source are shown in Figure 5. The experiments were conducted after the conclusions of the experiments with the 300 W lamp. The overall cell capacitance has increased for both the reference and light-exposed cases. The relative capacitance values were 13%, 15%, and 26% for blue, yellow and no-filter cases, respectively.

**Figure 6:**
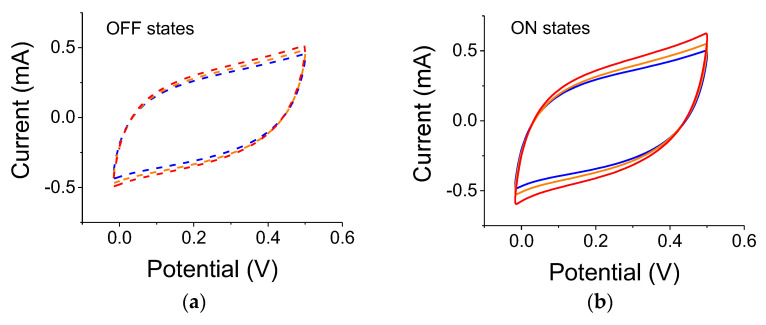
(**a**) Light OFF, C-V reference curves for 2 µm particles measured before illumination with a 75 W white-light source. Experiments were carried out after the conclusions of the experiments with the 300 W lamp. The colored dashed curves correspond to successive references for data taken with the blue, yellow, and no filter, respectively. (**b**) C-V curves for light ON cases; the blue, yellow, and red curves correspond to illumination with blue, yellow, and no filter, respectively. Scan rate was 0.01 V/s.

Similar experiments were conducted with 2 µm TiO_2_ particles. Relative capacitance changes with the 300 W white-light source are 24%, 19%, and 29% for blue, yellow, and no-filter cases, respectively. Using a 75 W white-light source, we obtained the results shown in Figure 6. The actual capacitance values for this particular sample are lower than the one used with the 0.2 µm particles probably due to inconsistency in film manufacturing. Nevertheless, the relative capacitance changes were 11%, 12%, and 19% for blue, yellow, and no-filter cases, respectively.

The results for a 3-electrode setting illuminated by a 300 W white-light source are summarized in Table 2. Some differences are noted for the ‘Blue’ and ‘No Filter’ cases yet, overall, the results portray a similar trend for both particle sizes.

The results for a 3-electrode setting illuminated by a 75 W white-light source are summarized in Table 3. There is a distinction between irradiation with and without filters. The larger response is obtained without any filter, whereas the light-induced response with either filter is similar despite the differences in their transmission values.

**Figure 7:**
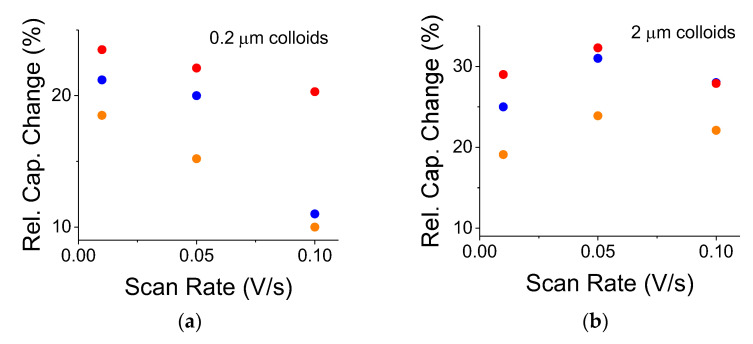
Comparison between relative capacitance changes, (C_light ON_−C_light OFF_)/C_light OFF_, as a function of scan rate for various illumination conditions. Red, yellow, and blue dots represent illumination, respectively, without a filter, through a yellow filter, and through a blue filter. (**a**) For embedded 0.2 μm colloids. (**b**) For embedded 2 μm colloids. The white light source was a 300 W incandescent lamp.

Using C-V, a typical double-layer S-C exhibits an exponential-like decreasing capacitance as a function of the increasing scan rate, mainly due to the slow, ionic diffusion response in the electrolyte. The TiO_2_-embedded capacitors exhibited a similar trend (not shown). Yet, the relative capacitance change may not necessarily follow such an exponential trend because its normalized expression factors out the ionic response. Although the smaller 0.2 μm colloids exhibited a weak, decreasing almost linear trend (Figure 7a), their larger counterparts, the 2 μm colloids, exhibited almost constant behavior (Figure 7b). The experiments were run from the faster scan rate toward the slower one to avoid residual cell polarization. The curve for the OFF states was repeated after each illuminated case.

**Figure 8:**
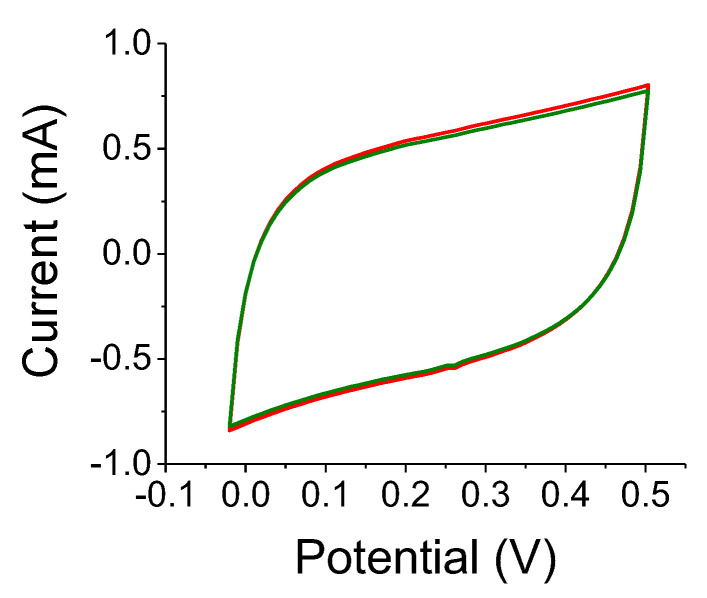
C-V curves for S-C without TiO_2_ colloids for an OFF, ON, OFF cycle. The scan rate was 0.1 V/s.

A reference experiment is shown in Figure 8. Results for the A-C electrode without the TiO_2_ colloids under 300 W white-light illumination are shown. The two OFF state curves (black and green) are indistinguishable, whereas the capacitance increase is assessed as being less than 3%. This ought to be compared to Figure 7 where the relative changes for 0.2 μm and 2 μm TiO_2_-embedded electrodes were 22% and 29%, respectively. The film thickness was relatively small, of the order of 150 μm and the experiments on two different samples yielded similar results.

**Figure 9:**
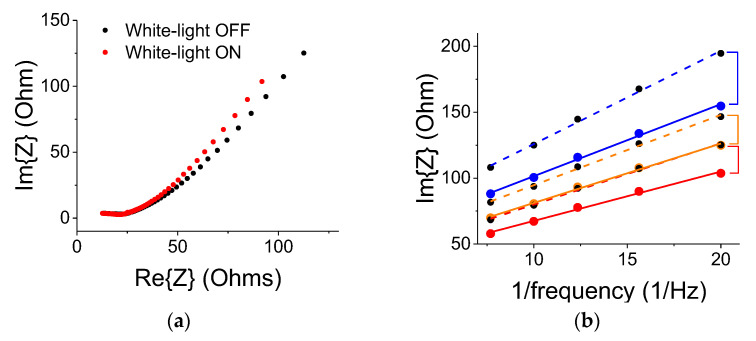
Results using EIS with a 300 W light source. (**a**) A typical EIS curve for 2 μm TiO_2_ particles with no filter. There is no light-induced change in the electrode resistance at Re{Z}~25 Ohms. (**b**) Analysis of the data from 5 mHz (20 1/Hz) up to 140 mHz (7.25 1/Hz) revealed a linear dependence on the inverse frequency and the lingering effect of prolonged illumination as measured in light OFF conditions. The colored dashed curves correspond to successive references (OFF states) for data taken with the blue, yellow, and no filter (red), respectively. The colored solid lines represent ON state data that were measured with blue, yellow, and no filter (red curve), respectively.

The C-V data were corroborated by electrochemical impedance spectroscopy (EIS) measurements [22]. The frequency range was from 50 mHz to 50,000 Hz and the amplitude of the modulating signal was 20 mV. As shown in Figure 9a, the white-light illumination by a 300 W lamp resulted in a small change to the electrode’s impedance (the region up to the curve’s knee); the electrode impedance change was less than 2%. It means that the optical contribution by the ITO electrode, which was facing the light source was rather minimal. Under the light exposure, the curve’s slope beyond the knee became larger, indicating a larger capacitive effect. The differential capacitance plots (Im{Z} vs. 1/frequency) are shown in Figure 9b. The blue, yellow, and red curves correspond to the ON states with blue, yellow, and no filter, respectively. The dashed colored curves correspond to successive references (OFF states) for data taken with the blue, yellow, and no filter, respectively. The curves were linear (as they should be for a double-layer capacitor) in the frequency region between 50 and 140 mHz (7.25 to 20 1/Hz region shown in the graph). The differential capacitance is inverse to the curve’s slope. The lingering polarization effect in the OFF state is exhibited by successive smaller slopes. The trend corroborates the results shown in Figure 6.

## 4. Discussion

A clear, light-induced capacitance increase has been observed for both 0.2 and 2 μm TiO_2_ particles that were embedded in A-C/PMMA electrodes. From Table 3, a similar, relative capacitance increase was recorded for both particle sizes under illumination by a 75 W light bulb. Additionally, both particle sizes portrayed a similar optical response when using blue and yellow filters. This points to the greater effect of the blue portion of the irradiation spectrum since the blue band constituted only 10% of the entire white-light intensity, whereas the light intensity transmitted by the yellow filter constituted 40% of the overall white-light intensity. When the filters were removed, the light-induced effect increased by more than 50% due to an increase in the light intensity and a wider blue band spectrum. On the other hand, when the light intensity increased fourfold (Table 2), the response was similar for both particle sizes and was quite similar when the filters were in or out of place. This suggests a light-saturation effect by the colloids; under the intense light, the colloids absorbed light to full capacity.

The colloidal size had an impact on the relative capacitance change under various scan rates. The capacitance change dropped at larger scan rates for the smaller colloids while remaining almost constant for the larger ones. For the larger colloids, this implies a slower optical response time. Typically, small TiO_2_ colloids exhibit optical lifetime on a scale of <1 ns [23]. On the other hand, photo-electrochemical lifetimes for compacted submicron TiO_2_ electrodes are on the scale of seconds [24]. Thus, it seems that micron-sized colloids exhibit even longer lifetimes: the electrode resistance did not change much upon illumination (Figure 9); Figure 7b indicates a capacitance peak at a scan rate of 0.05 V/s or a cycle of 20 s; therefore, the RC constant indicates an optically related capacitance change on a scale of 10 s of seconds. It is suggested that the photo-excited carriers were trapped, either by surface states or at defects at the polymer/colloid interface. The lingering polarization effect that was exhibited by the OFF states may also suggest such a trapping mechanism. Finally, optically enabled charge-trapping mechanisms may explain the lack of faradaic behavior, which is typical of pseudocapacitors, namely, the absence of chemical reaction peaks in the C-V curves - the effect is purely physical. Neither as-is A-C electrodes (Figure 8), nor other semiconductor embedded systems [20,21] seem to exhibit such a lingering polarization effect.

## 5. Conclusions

Light-induced capacitance increase was observed in S-C, whose A-C electrodes were embedded with TiO_2_ colloids. Data alluded to a saturation effect at large light intensities, the relatively greater impact of the blue portion of the illumination spectrum, and a seemingly small difference between the light response of 0.2 and 2 μm colloids to light intensity or color. The latter has practical implications because larger TiO_2_ are easier to make and are cheaper to buy. Larger colloids seem to exhibit a longer electrochemical lifetime. Lingering cell polarization after long irradiation times points to the formation of trapped carriers.

## Figures and Tables

**Table 1:** Relative capacitive change using C-V measurements with a 2-electrode setting when illuminated by a 75 W white-light source. Similar results were obtained using electrochemical impedance spectroscopy (EIS).

	Rel. Cap Change 0.2-µm TiO_2_	Rel. Cap Change 2-µm TiO_2_
Blue Filter	5%	6%
Yellow Filter	5%	6%
No Filter	11%	10%

**Table 2:** Relative capacitive change using C-V measurements in a 3-electrode setting when illuminated by a 300 W white-light source.

	Rel. Cap Change 0.2-µm TiO_2_	Rel. Cap Change 2-µm TiO_2_
Blue Filter	21%	24%
Yellow Filter	19%	19%
No Filter	23%	29%

**Table 3:** Relative capacitive change using a C-V measurements in a 3-electrode setting when illuminated by a 75 W white-light source.

	Rel. Cap Change 0.2-µm TiO_2_	Rel. Cap Change 2-µm TiO_2_
Blue Filter	13%	11%
Yellow Filter	15%	12%
No Filter	26%	19%

## Data Availability

Data is available upon reasonable request.

## References

[1] Yan J., Wang Q., Wei T., Fan Z. (2014). Supercapacitors: Recent advances in design and fabrication of electrochemical supercapacitors with high energy densities. Adv. Energy Mater..

[2] Kim B.K., Sy S., Yu A., Zhang J. (2015). Electrochemical Supercapacitors for Energy Storage and Conversion. Handbook of Clean Energy Systems.

[3] Okonkwo P.C., Collins E., Okonkwo E., Sadasivuni K.K., Ponnamma D., Kim J., Cabibihan J.J., AlMaadeed M.A. (2017). 18—Application of Biopolymer Composites in Super Capacitor. Biopolymer Composites in Electronics.

[4] Bu F., Zhou W., Xu Y., Du Y., Guan C., Huang W. (2020). Recent developments of advanced micro-supercapacitors: Design, fabrication and applications. npj Flex. Electron..

[5] Velasco A., Ryu Y.K., Boscá A., Ladrón-de-Guevara A., Hunt E., Zuo J., Pedrós J., Calle F., Martinez J. (2021). Recent trends in graphene supercapacitors: From large area to microsupercapacitors. Sustain. Energy Fuels.

[6] Miao X., Rojas-Cessa R., Mohamed A., Grebel H. The Digital Power Networks: Energy Dissemination Through a Micro-Grid. Proceedings of the IEEE 2018 International Congress on Cybermatics.

[7] Lee S., Park S. (2013). TiO_2_ photocatalyst for water treatment applications. J. Ind. Eng. Chem..

[8] Kumar R., Singh B.K., Soam A., Parida S., Sahajwalla V., Bhargava P. (2020). In situ carbon-supported titanium dioxide (ICS-TiO_2_) as an electrode material for high performance supercapacitors. Nanoscale Adv..

[9] Pham V.H., Nguyen-Phan T.-D., Tong X., Rajagopalan B., Chung J.S., Dickerson J.H. (2018). Hydrogenated TiO_2_@reduced graphene oxide sandwich-like nanosheets for high voltage supercapacitor applications. Carbon.

[10] Pant B., Park M., Park S.-J. (2019). TiO_2_ NPs Assembled into a Carbon Nanofiber Composite Electrode by a One-Step Electrospinning Process for Supercapacitor Applications. Polymers.

[11] Elmouwahidi A., Bailón-García E., Castelo-Quibén J., Pérez-Cadenas A.F., Maldonado-Hódar F.J., Carrasco-Marín F. (2018). Carbon–TiO_2_ composites as high-performance supercapacitor electrodes: Synergistic effect between carbon and metal oxide phases. J. Mater. Chem..

[12] Sharavath V., Sarkar S., Ghosh S. (2018). One-pot hydrothermal synthesis of TiO_2_/graphene nanocomposite with simultaneous ni-trogen-doping for energy storage application. J. Electroanal. Chem..

[13] Salari M., Aboutalebi S.H., Konstantinov K., Liu H.K. (2011). A highly ordered titania nanotube array as a supercapacitor electrode. Phys. Chem. Chem. Phys..

[14] Barai H.R., Rahman M.M., Joo S.W. (2017). Annealing-free synthesis of K-doped mixed-phase TiO_2_ nanofibers on Ti foil for electro-chemical supercapacitor. Electrochim. Acta.

[15] Selvakumar M., Bhat D.K. (2012). Microwave synthesized nanostructured TiO_2_-activated carbon composite electrodes for super-capacitor. Appl. Surf. Sci..

[16] Mohammadpour A., Shankar K. (2010). Anodic TiO_2_ nanotube arrays with optical wavelength-sized apertures. J. Mater. Chem..

[17] Macak J.M., Schmuki P. (2006). Anodic growth of self-organized anodic TiO_2_ nanotube in viscous electrolytes. Electrochim. Acta.

[18] Ramadoss A., Kim G.-S., Kim S.J. (2013). Fabrication of reduced graphene oxide/TiO_2_ nanorod/reduced graphene oxide hybrid nanostructures as electrode materials for supercapacitor applications. CrystEngComm.

[19] Grätzel M. (2003). Dye-sensitized solar cells. J. Photochem. Photobiol. C Photochem. Rev..

[20] Grebel H. (2021). Optically Controlled Supercapacitors: Functional Active Carbon Electrodes with Semiconductor Particles. Materials.

[21] Grebel H. (2021). Asymmetric Supercapacitors: Optical and Thermal Effects When Active Carbon Electrodes Are Embedded with Nano-Scale Semiconductor Dots. J. Carbon Res..

[22] Mei B.-A., Munteshari O., Lau J., Dunn B., Pilon L. (2017). Physical Interpretations of Nyquist Plots for EDLC Electrodes and Devices. J. Phys. Chem. C.

[23] Brüninghoff R., Wenderich K., Korterik J.P., Mei B.T., Mul G., Huijser A. (2019). Time-Dependent Photoluminescence of Nanostructured Anatase TiO_2_ and the Role of Bulk and Surface Processes. J. Phys. Chem. C.

[24] Calva-Yáñez J.C., de la Fuente M.S., Ramírez-Vargas M., Rincón M.E. (2018). Photoelectrochemical performance and carrier lifetime of electrodes based on MWCNT-templated TiO_2_ nanoribbons. Mater. Renew. Sustain. Energy.

